# Generation of Gradients on a Microfluidic Device: Toward a High-Throughput Investigation of Spermatozoa Chemotaxis

**DOI:** 10.1371/journal.pone.0142555

**Published:** 2015-11-10

**Authors:** Yi Zhang, Rong-Rong Xiao, Tailang Yin, Wei Zou, Yun Tang, Jinli Ding, Jing Yang

**Affiliations:** 1 Reproductive Medicine Center, Renmin Hospital of Wuhan University, Wuhan, Hubei Province, China; 2 Key Laboratory of Analytical Chemistry for Biology and Medicine (Ministry of Education), College of Chemistry and Molecular Sciences, Wuhan University, Wuhan, China; Universidad Nacional Autónoma de México, MEXICO

## Abstract

Various research tools have been used for *in vitro* detection of sperm chemotaxis. However, they are typically poor in maintenance of gradient stability, not to mention their low efficiency. Microfluidic device offers a new experimental platform for better control over chemical concentration gradient than traditional ones. In the present study, an easy-handle diffusion-based microfluidic chip was established. This device allowed for conduction of three parallel experiments on the same chip, and improved the performance of sperm chemotaxis research. In such a chip, there were six channels surrounding a hexagonal pool. The channels are connected to the hexagon by microchannels. Firstly, the fluid flow in the system was characterized; secondly, fluorescein solution was used to calibrate gradient profiles formed in the central hexagon; thirdly, sperm behavior was observed under two concentration gradients of progesterone (100 pM and 1 mM, respectively) as a validation of the device. Significant differences in chemotactic parameters were recognized between experimental and control groups (*p* < 0.05). Compared with control group, sperm motility was greatly enhanced in 1 mM group (*p* < 0.05), but no significant difference was found in 100 pM group. In conclusion, we proposed a microfluidic device for the study of sperm chemotaxis that was capable of generating multi-channel gradients on a chip and would help reduce experimental errors and save time in experiment.

## Introduction

In mammals, only few spermatozoa succeed in arriving at the oviduct after rounds of biological selection. The population of sperm which can fertilize the egg is even smaller [1, 2]. It seems inconvincible that sperm can successfully find the egg just by randomly swimming, without any guidance. Chemotaxis has been proposed to be a possible mechanism during the process of sperm guiding to oocytes within the oviduct [3]. It is defined as the oriented movement of sperm towards gradients of chemicals released from oocytes or cumulus cells. However, it remains to be an uncertain answer whether chemotaxis is a common phenomenon in mammalian species and what are the exact chemoattractants.

Before the introduction of microfluidic devices, tools that have been used for *in vitro* detection of sperm chemotaxis were usually poor in the ability to control and maintain chemical concentration gradients [4, 5]. Microfluidic devices, instead, can provide well-defined and stable gradients. Moreover, structure of a microfluidic chip can be flexibly designed to satisfy demands of different experiments. The outstanding advantages in miniaturization and high-throughput analysis also make it more economical and efficient.

In this article, we proposed an easy-handle diffusion chip that allows simultaneous observation of sperm behavior in three parallel experiments. In such a chip, there were six channels surrounding a hexagonal pool. The channels are connected to the hexagon by 5 μm wide cross-channels. Progesterone concentration gradients were established in the central hexagon and chemotactic responses were detected in the hexagon as sperm sensed the gradient of progesterone.

## Materials and Methods

### Design and fabrication of the microfluidic device

**Fig 1:**
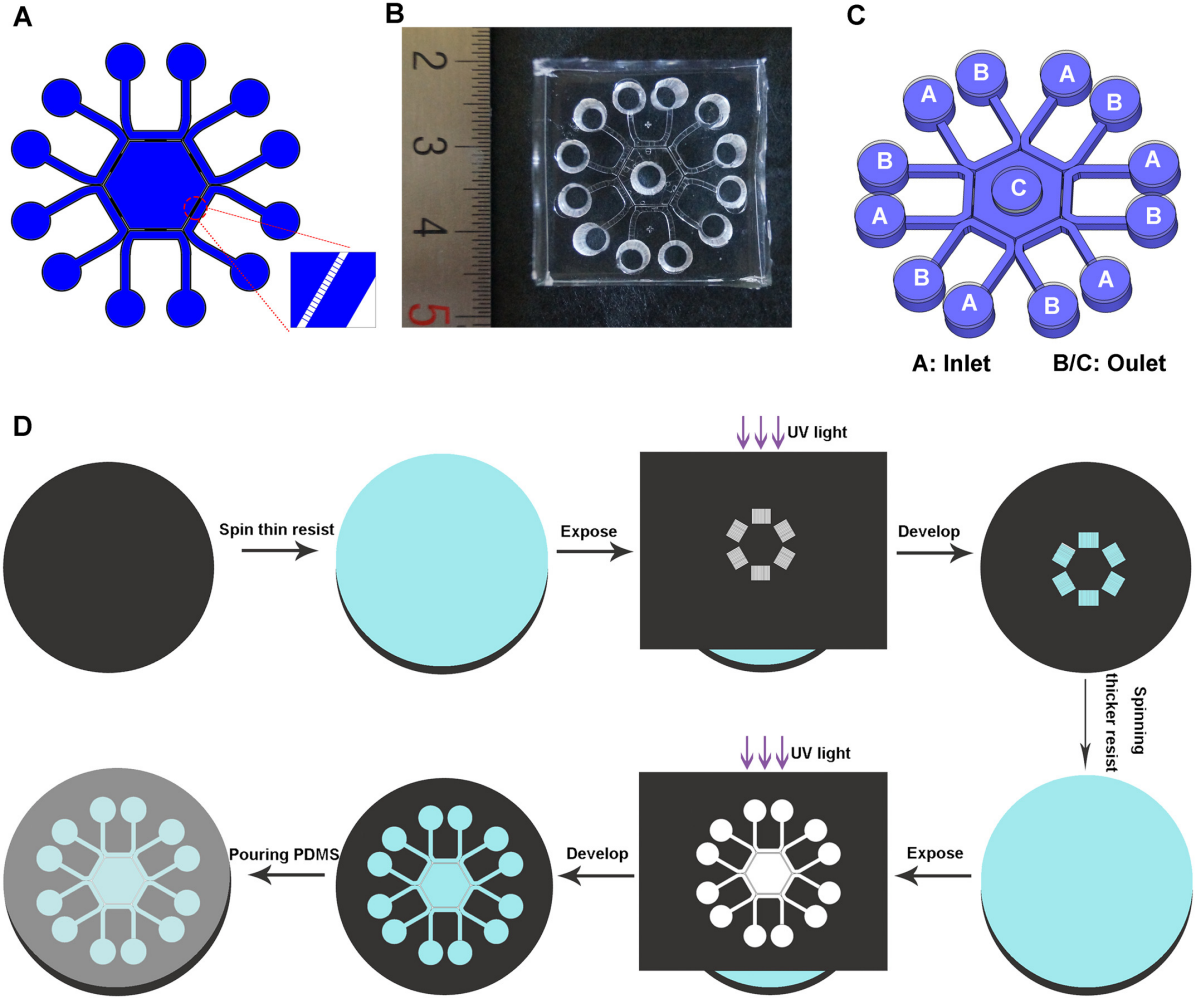
Design and fabrication of the microfluidic device. (A) Design of the microfluidic device. (B) A photograph of the chip. (C) A three-dimensional illustration of the device. Liquid level in Pool A was higher than that in both Pool B/C (details were listed in Table 1). (D) Fabrication process of the device (pictures were not drawn to scale).

The device was composed of a hexagon surrounded by six U-shape channels. Each side of the hexagon is connected with a peripheral channel by 15 microchannels (Fig 1A and 1B). Fluid flow in the device was controlled hydrostatically by adjusting the relative liquid level between the inlet and outlet pools (Fig 1C and Table 1). Dimensions of these channels were as follows: microchannels were 5 μm in width, 2 μm in height while other channels were 50 μm in height; peripheral channels were 700 μm wide; side length of the central hexagon was 4 mm. We used soft lithography technology to fabricate the chip [6–8]. Briefly, microchannels were patterned by spinning a thinner layer of SU-8 (2002) photoresist on a cleaned and dried silicon wafer. Then the wafer was exposed to UV light through a high-resolution mask. Other channels were patterned by spinning a thicker layer of SU-8 (2050) photoresist on the same silicon wafer which was already patterned with microchannels. The master was subsequently cured at 160°C for 30 min to further cross-link the material. PDMS mixture (oligomer/curing agent in a proportion of 10:1) was poured onto the master, cured at 75°C for 2 h (Fig 1D). Loading holes with the same diameter of 4 mm were punched in the chip after peeled off the master. The coverplate was molded by pouring PDMS mixture onto a clean petri-dish. Then two PDMS layers were treated in oxygen plasma (Harrick Scientific, Ossining, NY) for 1 min and bound together irreversibly.

**Table 1:** Initial volume of solution and hydrostatic pressure in each loading pool.

Loading volumes (μl)/Hydrostatic pressures (Pa)^a^			ΔH (μm)/ΔP (Pa)^b^
Loading plan	Pool A (inlet)^c^	Pool B/C (outlet)	
**P1**	30/23.41	20/15.61	800/7.8
**P2**	35/27.31	20/15.61	1200/11.7
**P3**	40/31.21	20/15.61	1600/15.6

^a.^ Hydrostatic pressure was calculated using the formula P = ρgh (ρ-the density of the solution, considered close to water; g-gravitational acceleration, 9.8 m/s^2^; h-liquid height in each pool). Liquid height in each pool was calculated using the formula V = *πr* ^2^ *h* (V refers to volume of solution added in each pool, diameter of each pool was 2 mm).

^b.^ ΔH/ΔP refers to liquid height/hydrostatic pressure differences between Pool A and B/C.

^c.^ Pool A, B and C were corresponded to the loading pools labeled in Fig 1C.

### Finite element simulation

A finite element analysis (COMSOL Multiphysics 3.5) software was used to simulate the fluid flow in the designed chip. A simpler 2D geometry which consisted of a hexagon surrounded by micro-channels and six peripheral channels was generated in COMSOL interface. Navier-Stokes equation for incompressible flow and convection-diffusion equation were used for simulations. Boundary conditions were laminar inflow at the inlets, laminar outflow at the outlets and no slip on the walls. The pressures applied in the inlets and outlets were listed in Table 1. Mesh density of the entire domain was set to extra fine. Stationary analysis was performed to reduce the time necessary for model calculating.

### Fluid flow characterization

The channels were primed with 1% phosphate buffered saline (PBS), containing 5% (w/v) bovine serum albumin (BSA) overnight and then flushed with Quinn’s^™^ Spermatozoa Washing Medium (SWM; SAGE, USA). 0.01% (w/v) solution of fluorescent microspheres (0.76 μm) (Sphere Scientific co., Wuhan, China) was utilized to characterize the fluid flow in the system. The characterization was carried out from two aspects. Firstly, one of the peripheral channels was doped with microspheres through both the inlet and outlet reservoirs. Other five channels as well as the central hexagon were loaded with SWM. Secondly, to further investigate the influence of liquid flow on sperm motility, microspheres were then added into the central hexagon while all the six peripheral channels were doped with SWM. Volumes of liquid pipetted into each loading pool were listed in Table 1. The motion of the microspheres was recorded every 500 ms using multifunctional automated inverted fluorescence microscopy (AxioObserver Z1 with camera, ZEISS, Germany). The flow speed in the device was obtained by analyzing the track of microspheres using NIH Image J with MTrack J plugin (http://rsbweb.nih.gov/ij/).

### Gradient profile calibration

Fluorescein (Sigma-Aldrich, USA; MW = 332 Da) dissolved in SWM was used to characterize concentration gradient for its similar molecular weight to progesterone (MW = 314 Da). Fluorescein solution was loaded into one peripheral channel. Other five channels and the hexagon were doped with SWM. Volumes of solution added in each pool were listed in Table 1. Fluorescence was recorded at an exposure time of 400 ms and fluorescence intensity profiles were analyzed in Image J. Time t = 0 was defined as the point right after sample loading.

### Sperm collection and capacitation

This study was approved by the Ethics Committee of Renmin Hospital, Wuhan University. Written consent was obtained from each patient before experiment. Semen samples were collected from 5 healthy individuals by masturbation after 3–5 d of abstinence. Only samples exhibiting normal seminal parameters were included [9]. Sperm were allowed to liquefy for 30–60 min at room temperature and processed with gradient centrifugation using Sperm Separation Media (SAGE, USA) to get highly motile sperm [10]. Procedures were carried out according to protocols attached to the product as previously described [11]. The resulting pellet was re-suspended in Quinn’s^™^ Spermatozoa Washing Medium (SWM; SAGE, USA). This medium is a modified HEPES-buffered Human Tubal Fluid (HTF) containing 5% (w/v) human serum albumin. Suspensions were then incubated under an atmosphere of 5% CO_2_ at 37°C for 4 h to induce capacitation [12].

### Sperm chemotaxis assay

Prior to the experiment, progesterone (Sigma-Aldrich, USA) was dissolved in Dimethyl sulfoxide (DMSO) as a stock solution and serially diluted with SWM to achieve final concentrations. Microfluidic device was primed and flushed with SWM. Sperm suspensions were adjusted to 10 × 10^6^ /ml with SWM. Concentration gradients of progesterone were generated as following: to avoid a mutual interference of concentration gradients generated from two nearby channels, progesterone solution was added into every other peripheral channel; the remaining three were doped with SWM. As control, SWM was added into all peripheral channels. Sperm suspension was gently added into the central hexagon. 20 μl sperm suspension from one sample was separately added into three groups: Group A, 100 pM progesterone solution was used to generate concentration gradients; Group B, 1 mM progesterone solution was added; Group C, control group with no progesterone added. In Group A and B, there were three regions in the hexagon where concentration gradients were established, corresponding to three peripheral channels where progesterone solution was loaded. Each region with a concentration gradient could be seen as an independent experiment. A video was captured in each region to record sperm movement at 15 fps for 3 sec. In Group C, three regions were randomly chosen to record sperm movement. In each video, 50 spermatozoa were analyzed. By playing videos back and forth frame-to-frame, we tracked the trajectories of the heads of motile spermatozoa in Image J software with MTrack J plugin.

Chemotactic response was evaluated using directionality-based assay. $\bar{\Delta Χ}$, %ΔX>0, %ΔX/|ΔY|>1 were calculated as previously described [13]. Briefly, $\bar{\Delta Χ}$ represented averaged net distance of a sperm head toward the direction of concentration gradient; %ΔX>0 referred to percentage of sperm traveled towards the gradient; %ΔX/|ΔY|>1 represented percentage of sperm with a greater parallel displacement to the chemical gradient over vertical displacement.

Chemokinetic response was assessed using actual curvilinear path (ACP), defined as the path a sperm traveled in recorded time; straight path (SP), referring to the straight-line distance between the first and last position of a sperm head; linearity of the trajectory (LIN), defined as SP/ACP.

### Statistical analysis

In chemotaxis assay, statistical analysis was conducted in SPSS software. Mean values were calculated and compared using two-way analysis of variance (ANOVA) followed by Fisher's Least Significant Difference (LSD) using SPSS software. A statistically significant difference was recognized when *p* < 0.05.

## Results

### Characterization of fluid flow in the chip

**Fig 2:**
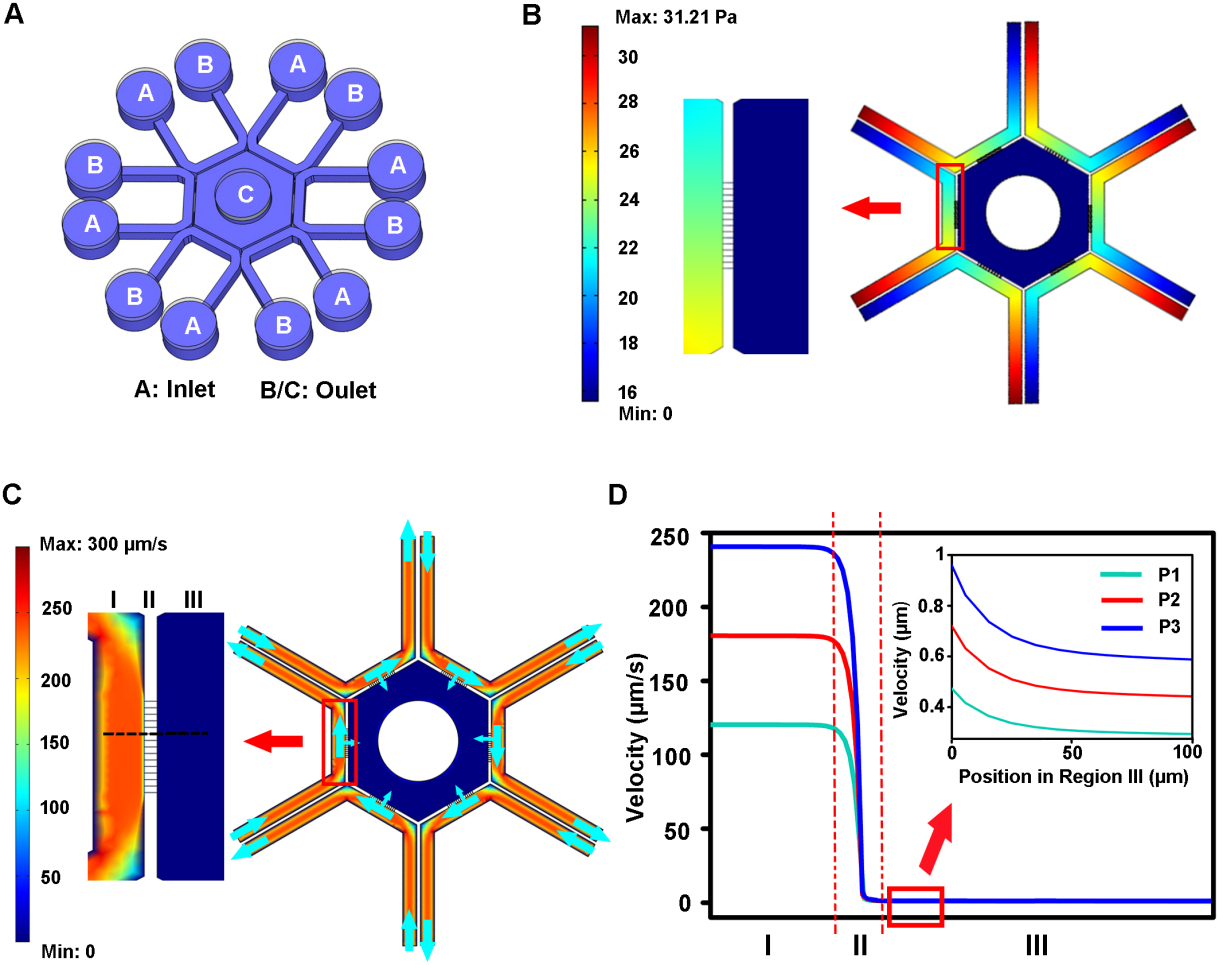
Simulation analysis of fluid flow in the device. (A) Schematic illustration of the microfluidic device. A and B/C represent the inlets and outlets of the channels, respectively. (B) Representative simulation of pressure distribution in the device. (C) Representative simulation of flow velocity distribution in the device. Blue arrow indicates the direction of fluid flow. (D) Distribution curves of flow velocity along the chip. I: peripheral channel; II: interconnecting grooves; III: central hexagon. Regions that were analyzed were indicated in (C) by a black dashed line. P1, P2 and P3 refers to different loading plans listed in Table 1.

A simplified model was established in COMSOL Multiphysics software. Pressures at the inlets and outlets were listed in Table 1. Since hydrostatic pressure existed between Pool A (inlet) and Pool B/C (outlet), fluid in Pool A could be driven to Pool B in the peripheral channel or to Pool C through micro-channels (Fig 2A). However, liquid flow from the peripheral channel to the hexagon was greatly restricted since the microchannels generated high liquid resistance to the flow. As depicted in Fig 2C and 2D, the flow speed in the peripheral channel is in the range of 100–250 μm/s. The speed was greatly reduced when the liquid flowed through the interconnecting grooves. The liquid flow was very weak in the central hexagon with a speed less than 1 μm/s. In addition, the flow speed within the device increased with the pressure difference between the inlets and outlets (Fig 2D). However, the influence of fluid inflow in the central hexagon on sperm movement was minor (S1 File).

**Fig 3:**
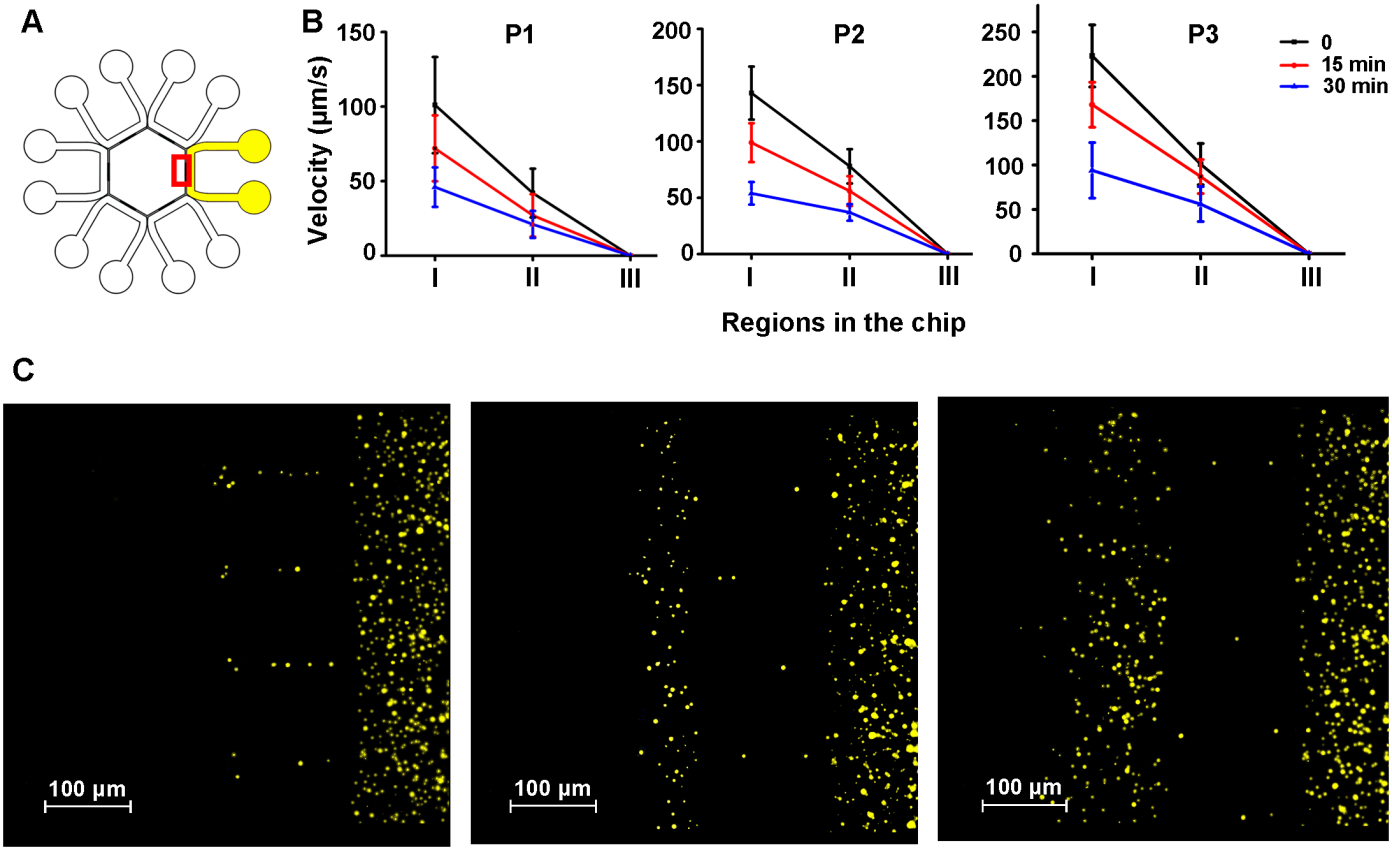
Characterization of fluid flow using microspheres. (A) Illustration of characterization experiment. Channels doped with microspheres were marked as yellow. The microsphere solution was added both in the inlet and outlet of the this channel. Other channels as well as the central hexagon were doped with SWM (marked as white). Red square indicated the region where (C) was captured. (B) The speed of microspheres in different region of the device (I: peripheral channel; II: interconnecting grooves; III: central hexagon) at 0, 15 min, 30 min after sample loading. P1, P2 and P3 were corresponded to the loading plans in Table 1. Data are presented as Mean ± SD (n = 5). (C) The distribution of microspheres in the device when liquid level in each pool reached equilibrium in different loading plans (P1, P2 and P3, successively).

To characterize the fluid flow in the device, solution of fluorescent microspheres was added in one of the peripheral channel while other five channels as well as the central pool was added with SWM (Fig 3A, volumes of solution were listed in Table 1). As liquid flowed from the inlets to the outlets, the liquid height differences between the loading pools were reduced. As a result, the flow velocity in the device was slowed down since the hydrostatic pressure in each pool was gradually equalized. The liquid level in each pool reached equilibrium in about 1 h, characterized by the absence of fluid flow in the channels. While there was an obvious flow in the peripheral channels, the flow in the central hexagon was minor, similar to the simulation analysis (Fig 3B and S1 Movie). Besides, only a small number of microspheres were found in the hexagon, indicating that the portion of liquid driven into the central hexagon from the peripheral channel was very small (Fig 3C). When ΔH was larger, more microspheres were driven into the central hexagon, indicating that the volume of solution driven into the hexagon was larger when higher hydrostatic pressure difference was applied (Fig 3C).

To further confirm the influence of fluid inflow on sperm movement in the central hexagon, solution of microspheres was only added in the central pool. In other loading pools, SWM was loaded (volumes of solution were listed in Table 1). Under three loading plans, only Brownian motion was observed among these microspheres which may be explained that the flow in the central hexagon was too minor to be observed. In any case, fluid inflow from the peripheral channels was very weak in the central hexagon, thus possessed little influence on sperm movement (S2 Movie).

### Generation of chemical concentration gradient

**Fig 4:**
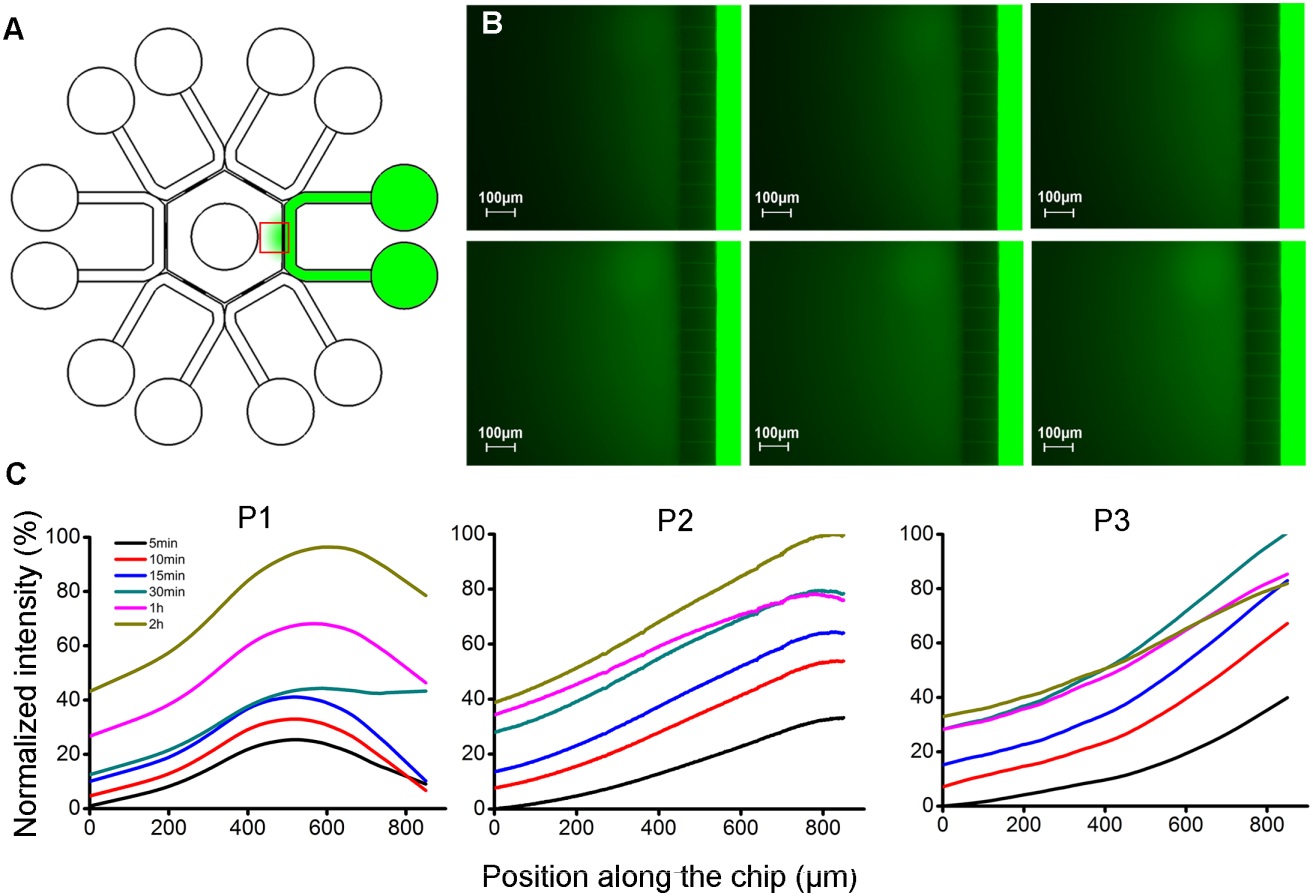
Gradient formation in the microfluidic device. (A) Fluorescein solution was doped into one channel and concentration gradient was formed in the hexagon (marked as green). Red square showed the area where fluorescence signals were recorded. (B) A representative fluorescence intensity change at 15 min, 30 min, 1 h, 2 h, 4 h and 7 h were displayed in sequence from left to right, top to bottom. (C) Normalized fluorescence intensity profiles in the central hexagon.

Profiles of concentration gradient varied with loading plans. Generally, in each plan, fluorescence intensity in the hexagon was enhanced as time proceeded while intensity in the peripheral channels barely attenuated. Quantitative analysis on the change of fluorescent signal in the hexagon was further carried out. In P1, no defined concentration gradient was established. In P2 and P3 where more significant liquid height differences were applied, gradients were well generated, characterized by fluorescence intensity positively correlated with positions in the hexagon, from center to side (Fig 4).

### Response of sperm to progesterone gradient

As showed above, concentration gradient could be generated either in P2 or P3. However, to avoid the impact of fluid flow on sperm migration as far as possible, P2 was chosen as the loading strategy in subsequent chemotaxis assays considering that the inflow speed in central hexagon is slower in P2 than in P3. In experimental groups (Group A and B), progesterone solution (100 pM or 1 mM) was added in the peripheral channels to generate concentration gradient. In control group (Group C), SWM was used. The volume of solution added in each loading pool was listed in Table 1.

**Fig 5:**
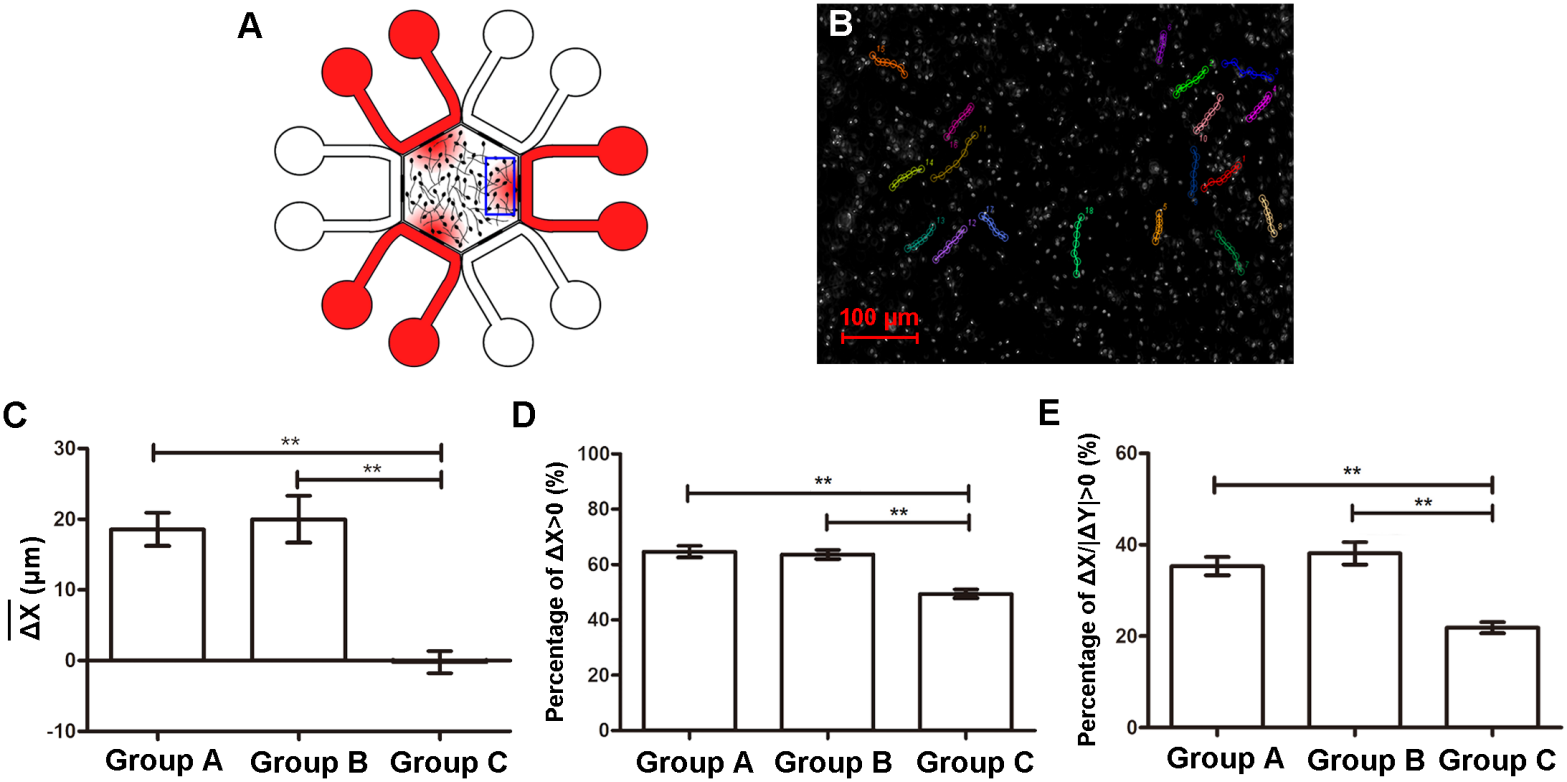
Chemotactic responses of spermatozoa to progesterone gradients. (A) An overview of concentration gradients generated in the hexagon. Progesterone solution was added in every other channel of the chip (red). Concentration gradients were generated in three regions in the hexagon corresponding to peripheral channels where progesterone solution was loaded. Blue square showed the field where sperm chemotaxis were observed. (B) A microscopic photograph of spermatozoa with several trajectories indicated (18 sperm). Each colored line represented a sperm trajectory within 3 s. (C-E) Comparisons of chemotactic parameters among three groups. Group A, 100 pM progesterone solution was added in peripheral channels; Group B, 1 mM progesterone solution was added; Group C, control. Data are presented as mean ± SD (n = 5). **: *p* < 0.05.

At fifteen minutes after sampling, the maximal progesterone concentration in the central hexagon was about 21 pM or 210 μM for the case when 100 pM or 1 mM were added into the peripheral channel respectively based on the fluorescein data. Behavior of the sperm were recorded. Indicators representing chemotactic response were analyzed (Fig 5; see S2 Fig for detailed data from each sample in each group). In Group A (100 pM progesterone solution was added in peripheral channels) and Group B (1 mM progesterone solution was added), the average net distances of sperm towards the concentration gradient ($\bar{\Delta Χ}$) were significantly higher than that in Group C (control group) (*p* < 0.05). Index %ΔX>0 were increased to 65% and 64% respectively in two experimental groups while this rate was only 49% in Group C (*p* < 0.05). This indicated that average percentage of chemotactic spermatozoa was about 16% in Group A and 15% in Group B, respectively. Compared with Group C, %ΔX/|ΔY|>1 were increased by 14% in Group A and 17% in Group B, respectively.

**Fig 6:**
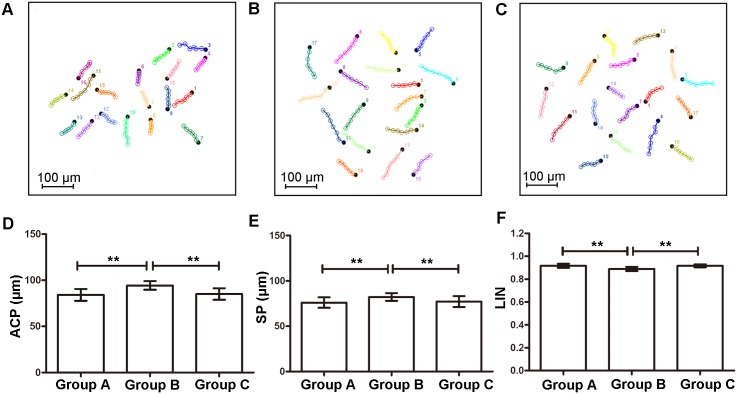
Chemokinetic response of spermatozoa to progesterone gradients. (A-C) Representative trajectories of sperm in Group A, B and C (18 sperm in each plot). Each colored line is a cell trajectory that is 3 s long. Black dots are the endpoints of the trajectories. (D-F) Different chemokinetic parameters were compared among three groups. Group A, 100 pM progesterone solution was added in peripheral channels; Group B, 1 mM progesterone solution was added; Group C, control. Data are presented as mean ± SD (n = 5). **: *p* < 0.05.

Chemokinetic response of sperm was quite different from chemotactic response (Fig 6; see S2 Fig for data from each sample in each group). In Group B, sperm motility, represented by ACP and SP, was greatly enhanced, accompanied by a decrease in linearity of sperm trajectories (LIN) (compared with Group A and C, *p* < 0.05). However, when lower concentration of progesterone (100 pM) was added (Group A), on the other hand, no significant difference was recognized in chemokinetic parameters (compared with Group C, *p* < 0.05).

## Discussion

In human beings, millions of spermatozoa are ejaculated into vagina, but only 1 in every million can reach the oviduct successively [14]. In order to figure out how such a small number of sperm could meet and fertilize the oocyte in the relatively vast space of oviduct, several guiding mechanisms have been proposed and chemotaxis is among one of them [15, 16].

In previous studies, many platforms have been proposed to investigate mammalian sperm chemotaxis [4, 16]. However, since only a small fraction of sperm were chemotactically responsive, all types of equipment were bothered with low signal-to-noise ratio. What’s more, sperm accumulation or concentration-dependent motility enhancement could also interfere with the observation of sperm chemotaxis. There are various types of assays applied in the study of mammalian sperm chemotaxis, but research tools used in those experiments usually lack an ability to generate a stable, controllable and reproducible concentration gradient. In addition, sizes of most tools are too large for a single micro-size cell, making it difficult to focus all cells on the same focal plane at the same time, which may interfere with sperm counting and assessment of sperm movement.

Compared with traditional devices, it is much easier to establish stable and controllable concentration gradients in a microfluidic chip. Gradient generators can be categorized as flow-based chips and flow-free (diffusion-based) chips [17]. Concentration gradient generated in a flow-based device is stable and long-time lasting, but the presence of fluid flow can greatly impact the sperm migration [18–21]. In a flow-free device, concentration gradient is formed mainly based on molecular diffusion. Currently, there are several diffusion-based chips used for chemotaxis. For example, Parthasarathy S. *et*. *al* established a diffusion chip to investigate the synergistic effects of 3D ECM and chemogradients on neurite outgrowth and guidance, however, this model may not be suitable for sperm since beating of flagella may be restricted due to the viscosity of hydrogels [22]. Another hydrogel-based chip proposed by Shing-Yi C. *et al*. was utilized for the study of sperm chemotaxis, but we thought the results would be more convincible if more sensitive indicators that represent sperm chemotaxis were evaluated [13, 23, 24].

In the present study, a gradient chip that allowed simultaneous conduction of three parallel experiments was proposed. Since the liquid flow into the central pool was very slow, and the amount of liquid flowing into the hexagon was small, the establishment of gradient in the central hexagon was mainly dependent on the diffusion effect. The effect of fluid flow on the sperm motility in the central pool could be neglected. As a validation of the device, response of human spermatozoa to progesterone was studied quantitatively. Three parallel experiments were conducted on a chip simultaneously, which saved time in experiment and helped reduce experimental errors. Sperm chemotaxis under two distinct progesterone concentrations (100 pM and 1 mM) were studied. In 100 pM group, a number of spermatozoa exhibited chemotactic behavior towards the concentration gradient, which was similar to previously reported [25, 26]. In 1 mM group, an obvious chemotactic response was also recognized, which was contrary to findings that cells did not chemotactically respond to progesterone gradient when the concentration was too high [25]. This can be explained from two aspects. Firstly, although the fluorescence intensity in the hexagon increased over time, it remained far weaker than that in the peripheral channel, and the fluorescence intensity of the periphery channel barely attenuated during the observation period. A quantitative analysis confirmed that the maximal progesterone in the central hexagon when sperm behavior was observed was 210 μM. This indicated that solution flowing into the central hexagon only accounted for very small proportion of that in the peripheral channel. Thus, when 1 mM of progesterone solution was added into the peripheral channel, the concentration in the hexagon was actually far lower than 1 mM. From this aspect, the concentration gradient in the hexagon may be in the range that chemotaxis could happen. Secondly, this intriguing phenomenon may be caused by temporal response to gradually increasing concentration of progesterone when receptors on sperm had not been saturated yet [27].

In addition to chemoattractive function, progesterone has been shown to have an impact on sperm motility as well. Chemokinematic parameters were further evaluated in order to investigate the concentration-dependent motility enhancement of sperm by progesterone. ACP and SP were greatly increased but linearity of sperm trajectories (LIN) was significantly decreased in 1 mM group, indicating that sperm were hyperactivated by a high concentration of progesterone. This was in accordance to previous studies[25, 28].

Progesterone was found to be associated with many biological behaviors of sperm [26, 29–31]. Given the importance of progesterone, it may be possible to assess sperm quality and recruit a subpopulation of good-quality spermatozoa utilizing sperm response to progesterone. Gatica *et al*. once carried out sperm selection assay based on the chemoattractive effect of progesterone [32]. In their study, spermatozoa enriched through the assay were in better physiological state for fertilization. In the present study, feasibility of our device in chemotaxis study has been approved. In further exploration, dimension of the chip may be adjusted to achieve high-efficiency collection of functional spermatozoa whose physiological features are much similar to sperm recruited from a natural selection process. Realization of this will inevitably contribute to improvement in efficiency and safety of assisted reproductive technology (ART). In addition, in view of the strong plasticity of microfluidic device, it may be possible to integrate more channels into the chip to simultaneously generate concentration gradients in more regions. Moreover, this chip may be integrated with other operational units for in vitro fertilization, leading to improved performance of ART.

## Supporting Information

S1 Fig Verification of the adsorption of microspheres onto the channels. Microsphere solution was added in one of the peripheral channels while other five channels as well as the central hexagon was doped with SWM. (A and C) The distribution of microspheres when liquid level reach equilibrium in each loading pool. (B) Liquid in one pool of the peripheral channel where microspheres were added was pipetted out after liquid level in each loading reservoir reached equilibrium. Particles in the peripheral channel were obscure since there was a flow of microspheres in the channel. (D) Liquid in the central pool was pipetted out and a large quantity of microspheres were driven from the peripheral channel into the hexagon.
(TIF)

S2 Fig Data of chemotactic response (A-C) and chemokinetic response (D-E) from each sample in each group. Group A, 100 pM progesterone solution was added in peripheral channels; Group B, 1 mM progesterone solution was added; Group C, control. Each column represents the mean ± SD of three parallel experiments from one sample.
(TIF)

S1 File The possible impact of fluid flow on sperm motility in the central hexagon. The text described the detailed calculation of the possible impact of fluid flow on sperm motility in the central hexagon.
(DOC)

S1 Movie Representative video of fluid flow in the device. Microspheres were driven from the peripheral channel into the central hexagon. There was a relatively fast flow speed in the peripheral channel. In the central hexagon, the flow was very weak and only Brownian movement could be recognized. The video is played in real time at 2 fps. Duration of the video is 10 s.
(MPG)

S2 Movie Movement of microspheres in the central hexagon. The distribution of microspheres were not influenced by the inflow of fluid from the peripheral channel since only Brownian movement was observed in the central hexagon. The video is played in real time at 2 fps. Duration of the video is 10 s.
(MPG)
