# Supplementary figures and images for: Generation of Gradients on a Microfluidic Device: Toward a High-Throughput Investigation of Spermatozoa Chemotaxis
Source: PLoS One. 2015 Nov 10;10(11):e0142555. doi: 10.1371/journal.pone.0142555 (PMC4640579; doi:10.1371/journal.pone.0142555)

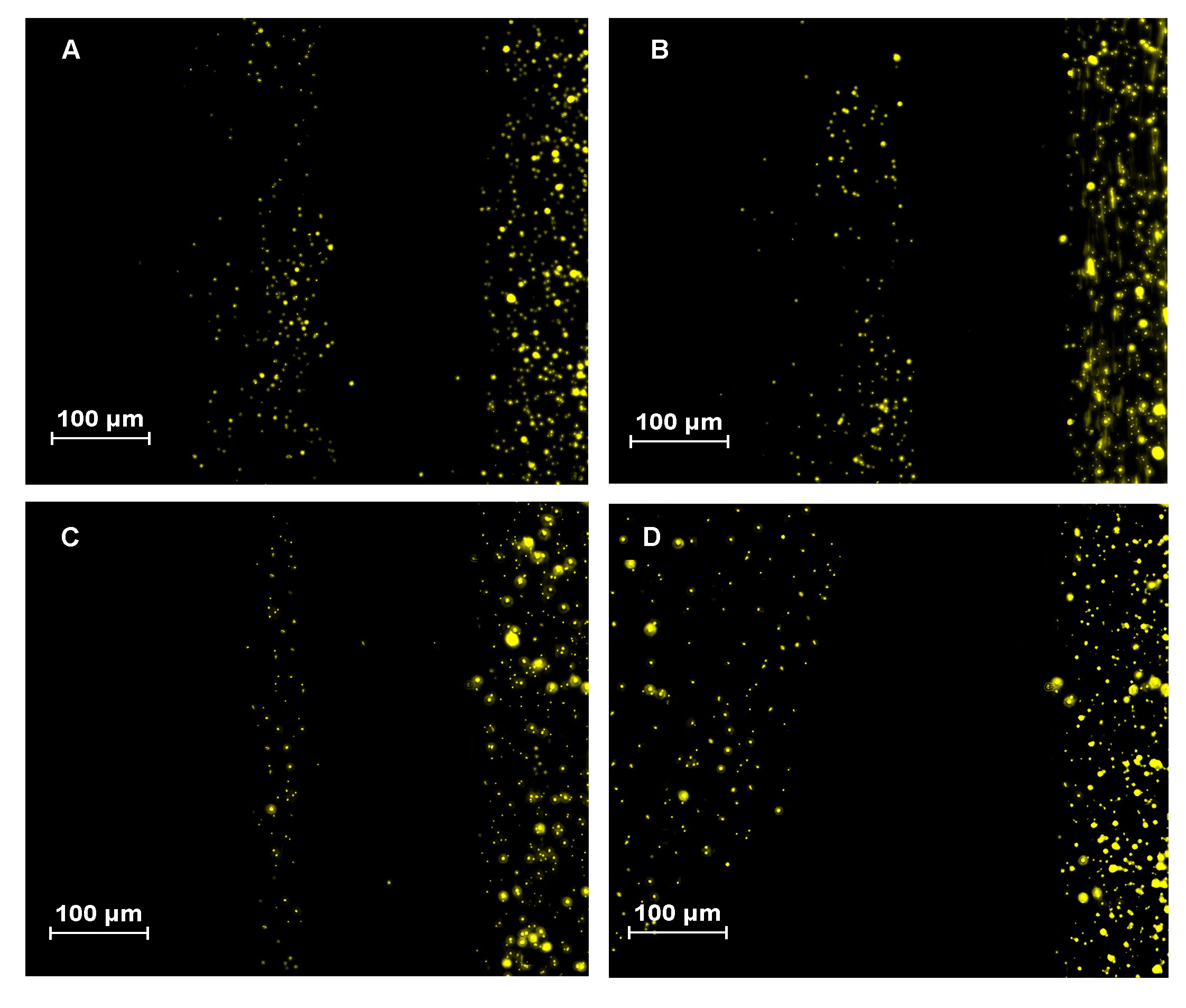

Supplement: S1 Fig — Microsphere solution was added in one of the peripheral channels while other five channels as well as the central hexagon was doped with SWM. (A and C) The distribution of microspheres when liquid level reach equilibrium in each loading pool. (B) Liquid in one pool of the peripheral channel where microspheres were added was pipetted out after liquid level in each loading reservoir reached equilibrium. Particles in the peripheral channel were obscure since there was a flow of microspheres in the channel. (D) Liquid in the central pool was pipetted out and a large quantity of microspheres were driven from the peripheral channel into the hexagon. (TIF) [file pone.0142555.s001.tif]

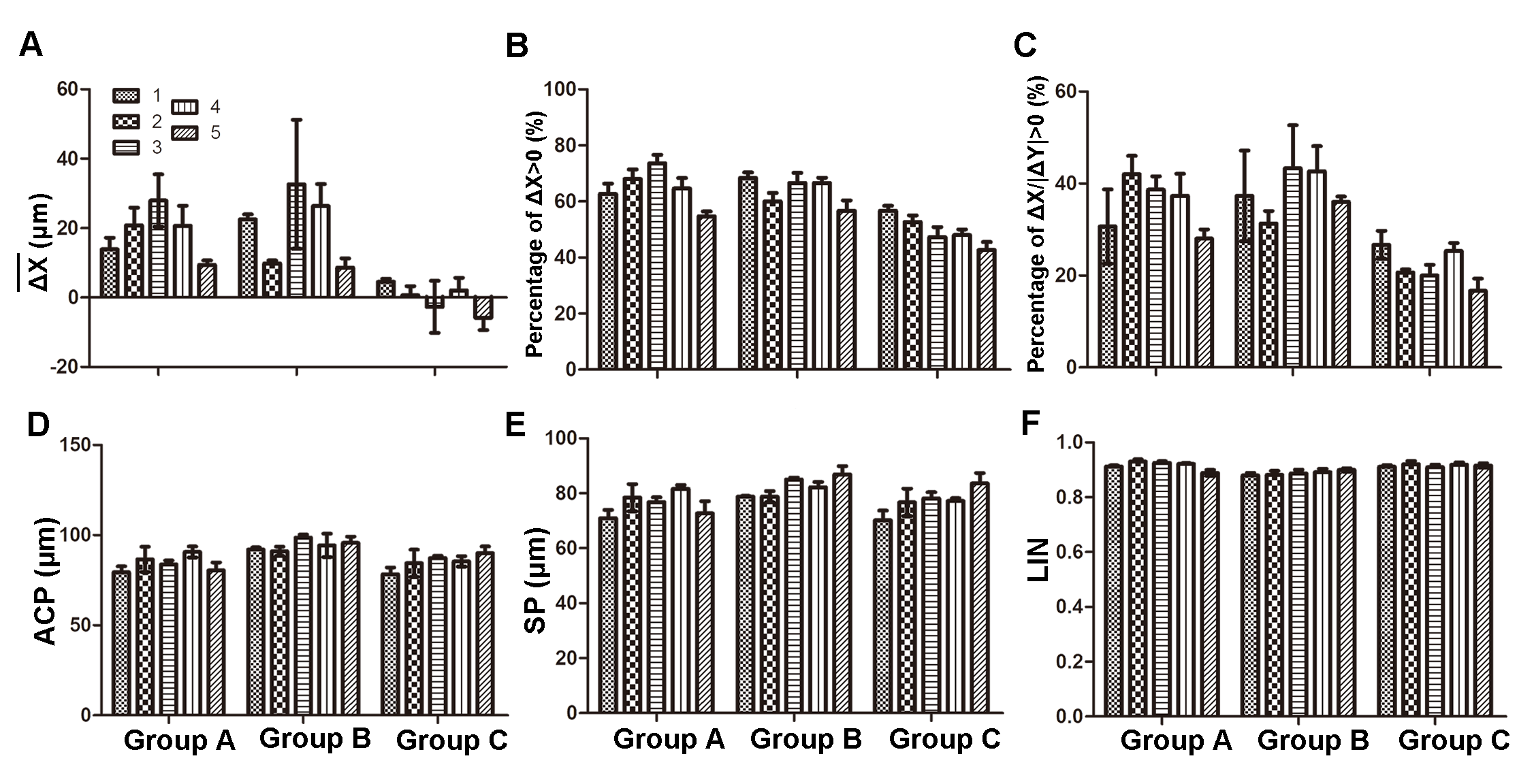

Supplement: S2 Fig — Group A, 100 pM progesterone solution was added in peripheral channels; Group B, 1 mM progesterone solution was added; Group C, control. Each column represents the mean ± SD of three parallel experiments from one sample. (TIF) [file pone.0142555.s002.tif]
