# Supplementary material for: Generation of Gradients on a Microfluidic Device: Toward a High-Throughput Investigation of Spermatozoa Chemotaxis
Source: PLoS One. 2015 Nov 10;10(11):e0142555. doi: 10.1371/journal.pone.0142555 (PMC4640579; doi:10.1371/journal.pone.0142555)
Supplement: S1 File — The text described the detailed calculation of the possible impact of fluid flow on sperm motility in the central hexagon. (DOC) [file pone.0142555.s003.doc]

**The influence of fluid inflow in the central hexagon on sperm movement**

The influence of fluid flow on sperm movement can be evaluated by the acceleration the flow posed to a single sperm by using the formula:

F=m_s_a⋅⋅⋅⋅⋅⋅⋅⋅⋅⋅⋅⋅⋅⋅⋅⋅⋅⋅⋅⋅⋅⋅⋅⋅⋅⋅⋅⋅⋅⋅⋅⋅⋅⋅⋅⋅⋅⋅⋅⋅⋅⋅⋅⋅⋅⋅⋅⋅⋅⋅⋅⋅⋅⋅⋅⋅⋅⋅⋅⋅⋅⋅⋅⋅⋅⋅⋅⋅⋅(1)

F-the force fluid flow generated to a single sperm; m_s_-the mass of a single cell; a-acceleration the fluid flow posed to the sperm.

Meanwhile, since the force was generated by the flow of the solution, the force could be calculated using the formula:

Ft=m_w_ν⋅⋅⋅⋅⋅⋅⋅⋅⋅⋅⋅⋅⋅⋅⋅⋅⋅⋅⋅⋅⋅⋅⋅⋅⋅⋅⋅⋅⋅⋅⋅⋅⋅⋅⋅⋅⋅⋅⋅⋅⋅⋅⋅⋅⋅⋅⋅⋅⋅⋅⋅⋅⋅⋅⋅⋅⋅⋅⋅⋅⋅⋅⋅⋅⋅⋅⋅⋅⋅(2)

t-a certain time period; m_w_-the mass of solution flowing into central hexagon through one interconnecting groove which posed force on a sperm; ν-the flow speed (the speed was assumed as uniform in the central hexagon).

m_w_ could be estimated by the formula:

m_w_=ρSνt⋅⋅⋅⋅⋅⋅⋅⋅⋅⋅⋅⋅⋅⋅⋅⋅⋅⋅⋅⋅⋅⋅⋅⋅⋅⋅⋅⋅⋅⋅⋅⋅⋅⋅⋅⋅⋅⋅⋅⋅⋅⋅⋅⋅⋅⋅⋅⋅⋅⋅⋅⋅⋅⋅⋅⋅⋅⋅⋅⋅⋅⋅⋅⋅⋅⋅(3)

ρ-density of the solution (assumed close to the density of water, i.e. 1 × 10^3^ kg/m^3^); S-the cross-sectional area of an interconnecting groove (2 μm × 5 μm, i.e. 1 × 10^-11^ m^2^).

Therefore, in Equation (2), m_w_ could be replaced by Equation (3), i.e. Ft=ρSνt (t can be removed on both sides of the equation). As a result, the resistance force could be expressed as:

F=ρSν^2^⋅⋅⋅⋅⋅⋅⋅⋅⋅⋅⋅⋅⋅⋅⋅⋅⋅⋅⋅⋅⋅⋅⋅⋅⋅⋅⋅⋅⋅⋅⋅⋅⋅⋅⋅⋅⋅⋅⋅⋅⋅⋅⋅⋅⋅⋅⋅⋅⋅⋅⋅⋅⋅⋅⋅⋅⋅⋅⋅⋅⋅⋅⋅⋅⋅⋅⋅⋅(4)

Thus Equation (1) could be written as ρSν^2^= m_s_a and the acceleration (a) could be calculated:

a=ρSν2/m_s_⋅⋅⋅⋅⋅⋅⋅⋅⋅⋅⋅⋅⋅⋅⋅⋅⋅⋅⋅⋅⋅⋅⋅⋅⋅⋅⋅⋅⋅⋅⋅⋅⋅⋅⋅⋅⋅⋅⋅⋅⋅⋅⋅⋅⋅⋅⋅⋅⋅⋅⋅⋅⋅⋅⋅⋅⋅⋅⋅⋅⋅⋅⋅⋅(5)

If the sperm is near the outlet of the interconnecting grooves and moves in the opposite direction of the fluid flow, the force on the sperm generated by the fluid flow would be the greatest. The simulation analysis revealed that the flow speed in the central hexagon was no more than 1 μm/s (1 × 10^-6^ m/s).

For estimation, the value of ρ is assumed as 1 × 10^3^ kg/m^3^, S is 1 × 10^-11^ m^2^ and ν is assumed as 1 × 10^-6^ m/s). The mass of a single sperm (m_s_) is assumed as 2.2×10^−14^ kg (mass calculated from volume assuming density of 1 g/mL)[1]. Thus “a” was calculated. The estimated value of “a” was 4.55 × 10^-7^ m/s^2^ (about 0.5 μm/s^2^).

In actual situation, the average speed of sperm from a healthy individual was around 35 μm/s. What’s more, in actual situation, the flow velocity would become slower and slower as the hydrostatic pressure difference between the inlets and outlets were reduced. In the chemotaxis assay, the observation of sperm motility was carried out in about 15 min after sample loading, at which time the flow speed would be much slower compared with that when solution was just added into the device. Therefore, the acceleration generated by the fluid flow would be much smaller than the estimated value of 0.5 μm/s^2^ and possessed little influence on sperm movement. Besides, since the flow in the central hexagon was extremely weak, the generation of concentration gradient was mainly based on the diffusion of molecules.

**References**

1. Curry MR, Millar JD, Tamuli SM, Watson PF. Surface area and volume measurements for ram and human spermatozoa. Biol Reprod. 1996;55(6):1325-32.
